# Utility of First Trimester Ultrasonography before 11 Weeks of Gestation: A Retrospective Study

**DOI:** 10.5402/2012/308759

**Published:** 2012-10-14

**Authors:** Sevki Celen, Necmiye Dover, Berna Seckin, Ufuk Goker, Okan Yenicesu, Nuri Danisman

**Affiliations:** Zekai Tahir Burak Women's Health Education and Research Hospital, Hamamonu, 06230 Ankara, Turkey

## Abstract

We showed the utility of first trimester ultrasonography before 11 weeks of gestation for antenatal followup. We retrospectively analyzed 1295 records of patients who underwent first trimester ultrasonography (transvaginal/abdominal) in our antenatal clinic in Ankara, Turkey. Maternal age, parity, gestational age, and maternal gestational history were compared with ultrasonographic findings. Patients were divided into 12 groups based on ultrasonographic diagnoses in the first ultrasonographic scan, and called for a control examination within 10 days if the diagnostic findings were abnormal. The data were statistically analyzed using Kruskal-Wallis and chi-square tests. We noted 81.3% patients to have single, viable, intrauterine pregnancies, while 18.7% had abnormal or complicated pregnancies with uterine anomalies, ovarian cysts, fibroids, or subchorionic hematomas. Normal and anembryonic pregnancies had significantly lower median diagnostic period in the control ultrasonography than in the first examination. First trimester ultrasonography before 11 weeks of gestation is valuable in determining pregnancy outcomes.

## 1. Introduction

The first trimester of pregnancy is one of the most fascinating periods of human development [1]. However, it is fraught with a high complication rate [2]. The last menstrual period (LMP) is generally used as a landmark for pregnancy dating, and the first trimester of pregnancy is defined as 12 weeks after the LMP [1]. First trimester ultrasonography aims to visualize viability, establish pregnancy dating, detect multiple pregnancy, observe uterine adnexal structures, measure nuchal translucency, evaluate foetal gross anomaly, and detect other special indications. 

To adequately assess first trimester pregnancy, the gestational sac (GS) size or embryonic crown rump length (CRL) should be compared with the menstrual age. An intrauterine GS is the first landmark consistently observed on ultrasonography in early pregnancy. The GS can be visualized as early as 4.5 weeks by the transvaginal technique [3, 4]. Anembryonic pregnancy is a form of failed pregnancy defined as a GS in which the embryo fails to develop. The secondary yolk sac (simply termed yolk sac) is the first structure to become visible within the GS [2, 5].The second structure that becomes sonographically visible within the GS is the embryo, which should be observed transvaginally when the GS measures ≥18 mm, and transabdominally when the GS measures ≥25 mm [5, 6]. 

By 5 weeks of gestation, the number of GSs within the uterus can be accurately counted. This “chorionic sac count” is merely the beginning of the diagnostic process and can predict only the chorionicity of multifetal pregnancies, if any [7]. In multiple gestation, ultrasonography examination should include examination of number of foetuses; confirmation of life; CRL and/or biparietal diameters; chorionicity or amnionicity; and if expertise is available, nuchal translucency assessment [8]. 

Subchorionic haemorrhage is defined as bleeding resulting in marginal abruption with separation of the chorion from the endometrial lining [9]. The majority of subchorionic haemorrhages occur in the late first trimester [10]. No definite correlation between haemorrhage size and pregnancy loss has been confirmed [11, 12]. 

Careful investigation of the uterus and adnexae is recommended as part of the routine first trimester evaluation. Myomas can grow during pregnancy and obstruct the birth canal [13]. Further, uterine duplication anomalies and septate uterus are associated with a high pregnancy loss rate [14]. 

In this retrospective study, we examined the diagnostic utility of the first antenatal ultrasonographic examination in first trimester pregnancies in the Zekai Tahir Burak Women's Health Education and Research Hospital Antenatal Clinic. We aimed to demonstrate the diagnostic value of early first trimester ultrasonography, especially before 11 weeks of gestation. 

## 2. Materials and Methods 

The records of pregnant patients who had undergone first trimester ultrasonography scanning in their first antenatal visit in Zekai Tahir Burak Women's Health Education and Research Hospital Antenatal Clinic, during January–December 2009, were retrospectively reviewed. The eligibility criterion was ultrasonographic findings of patients' first antenatal visit and a confirmatory control ultrasonographic scan. The patients were selected by a simple randomized method. Positive serum *β*hCG level above 1500 IU/mL was used as the criterion for undergoing ultrasonography. Pregnant patients with chronic metabolic diseases and known genital tract pathology or lesions were excluded. Written informed consent was obtained from all the eligible patients. When a patient showed a positive *β*hCG test result but did not have confirmed viable pregnancy, she was called for a second control ultrasonography within 10 days. 

Ultrasonographic examinations were performed by an obstetrics and gynaecology specialist in the antenatal polyclinic room with GE Logiq A5 convex probe (3.5 Hz) and transvaginal probe (5 Hz).

Age, parity, gestational age, special features regarding maternal gestational history—such as Rh-Rh isoimmunization, ultrasonographic findings, CRL or GS diameter, and foetal cardiac activity, as well as the presence/absence of subchorionic hematoma (SCH), multiple pregnancy, anembryonic pregnancy, adnexal mass, ectopic pregnancy, leiomyoma, and/or uterine anomalies were noted in the ultrasonographic examination. The patients were divided into 12 groups according to their ultrasonographic diagnosis. The groups were normal, anembryonic, multiple, and ectopic pregnancies; normal and anembryonic pregnancies in control ultrasonography; intrauterine ex fetus; subchorionic hematoma; impaired gestational sac; uterine anomaly; and fibroids and ovarian cysts with viable pregnancy.

All anembryonic pregnancies (in the first or control second ultrasonography) were diagnosed by the transvaginal technique using a 5-Hz probe. A mean GS diameter >20 mm (transvaginal technique) without a visualized embryo was considered as an anembryonic pregnancy. 

Statistical analysis was used to compare the diagnostic value of the first trimester ultrasonography. Kruskal-Wallis test was used for comparing the patients' median ages and median LMPs of time periods of diagnosis, and chi-square test was used for comparing the percentage of ultrasonographic diagnosis and their distribution according to patients' ages. *P* < 0.05 was considered statistically significant. 

## 3. Results

We analyzed 1295 first trimester pregnancy ultrasonography findings. Of these, 67.5% (*n* = 874) patients had a single, viable, intrauterine pregnancy in the first ultrasonography, while 13.9% (*n* = 180) patients had a viable, single intrauterine pregnancy on the control second ultrasonography but not in the first scan. Thus, 81.3% of our patients had an ultrasonographic single uncomplicated viable pregnancy diagnosis.  Table 1 shows the overall percentage of different ultrasonographic diagnostic findings and their distribution according to age groups (>35 and ≤35 years). Anembryonic pregnancies, degenerated GS, and intrauterine ex fetus diagnosis are defined as “abnormal pregnancies”. Viable single pregnancies with fibroids, ovarian cysts, subchorionic hematomas, and uterine anomalies are defined as “complicated pregnancies.” We determined the median ages and median gestational week (days) at the time of diagnosis for each group; the median gestational week at which a normal pregnancy was diagnosed was 56 days (minimum, 28 days; maximum, 85 days). The median ages at which normal pregnancy, anembryonic pregnancy, and intrauterine ex-pregnancy were diagnosed in the first ultrasonography were 24, 24, and 23 days, respectively. No statistical difference was noted among these three groups for the time of diagnosis (*P* = 0.651;  *P* > 0.05) (Table 2).

Normal pregnancies were diagnosed in the first ultrasonography within 56 days (median) from the LMP, while normal pregnancies in the control second ultrasonography were diagnosed within 42 days (median) from the LMP; the difference between the two groups was statistically significant (*P* = 0.0001,  *P* < 0.05). We consider that this difference occurred since all patients with potential but unconfirmed diagnosis of normal pregnancy were called for a control second ultrasonography within 10 days of the first ultrasonography. Similarly, anembryonic pregnancies were diagnosed in the first ultrasonography within 49 days (median) from the LMP, while anembryonic pregnancies were diagnosed in the second control ultrasonography within 45 days (median value); the difference was again significant (*P* = 0.0001,  *P* < 0.05). The presence of any suspicious diagnosis led to a lower median diagnostic period from the LMP. The diagnosis of intrauterine ex-fetus had the longest diagnostic period from the LMP, that is, 64 days (median); this was statistically different from all the other diagnostic groups (Table 3).

## 4. Discussion

The embryonic period lasts for 8 weeks after conception or 10 weeks after the LMP. This is the period of organogenesis, and most malformations are known to arise in this period [15]. During the 3rd and 5th weeks of gestation, fertilization occurs and the conceptus develops [1]. Worldwide, first trimester ultrasonography between 11 and 14 weeks of gestation is considered a valuable tool for early detection of foetal defects and aneuploidies. Nicolaides et al. have described the utility of measuring foetal nuchal translucency (NT) in 11–14 weeks ultrasonographic scans of pregnancies [16], while Cicero et al. determined that the incidence of absent nasal bone at 11–14 weeks is related to presence or absence of chromosomal defects [17]. These studies demonstrate the diagnostic value of 11–14-week ultrasonography for determining foetal health. However, our study focused on the diagnostic value of first trimester ultrasonography in our centre's antenatal clinic before 11 weeks of gestation. Here, we determined abnormalities or complications in 18.7% of pregnancies—such as uterine anomalies, ovarian cysts, fibroids, or SCH—before a mean gestational age of 64 days (approximately 9 weeks of gestation). 

Despite the use of ultrasonography to determine foetal or maternal pathology, the measurement of the CRL in the first trimester is the most accurate way to establish gestational age [18]. The first-trimester scan is even more beneficial in special cases of multiple gestations. The first trimester is the best time to establish chorionicity and amnionicity. In a dichorionic gestation, the dividing membrane shows a typical thickening (“lambda” or “twin peak” sign) as it approaches the placental surface [19, 20]. The mean gestational age (days) at which multifoetal pregnancies were diagnosed in our study was 51 days, while the incidence was 1.07% (12 of 1113 pregnancies); 83% (10 of 12 multiple pregnancies) of these multifoetal pregnancies comprised twins. We compared our findings with a retrospective study from Turkey, where Sezer et al. [21]. determined that the mean multiple pregnancy rate in Turkey was 1.9% and the mean twin birth rate was 1.7%. The large majority (80–97.3%) of multiple pregnancies in Turkey are twin pregnancies [18]. The multiple pregnancy rate in our centre differed from that previously reported in Turkish literature. This may be because our centre has a separate clinic for high-risk pregnancies where multiple pregnancies are followed. However, the twin pregnancy rate was in agreement with that previously reported. 

The value of ultrasonography in ectopic pregnancy diagnosis has been demonstrated repeatedly [22, 23]. In recent years, the incidence of ectopic pregnancy has increased, and although only approximately 1% of gestations are extrauterine, these account for 4% of direct maternal deaths [24]. In the presence of established risk factors or clinical suspicion for ectopic pregnancy, early ultrasonography is recommended [25, 26]. In our study, the incidence of ectopic pregnancy was 0.6%. In the literature, the incidence of ectopic pregnancy is 1.97 per 100 pregnancies [6]. However, in our centre, the examination for ectopic pregnancies is also performed by the emergency clinic. We did not consider ectopic pregnancies diagnosed by the emergency clinic in this patient sample, which may explain the lower rate of ectopic pregnancies. Further, all patients with ectopic pregnancy were <35 years. An age-adjusted ectopic pregnancy incidence study from Norway revealed the rate of ectopic pregnancies to be the highest among women aged 25–34 years [24]. This was compatible with our results [27].

Nagy et al. evaluated the long-term clinical significance of intrauterine hematomas detected in the first trimester of pregnancy in a general obstetric population, where the incidence was 3.1% [28]. Retroplacental hematomas were significantly correlated with an increased risk for adverse maternal and neonatal complications. Their results also indicated that the presence of an intrauterine hematoma during the first trimester may identify a population of patients at an increased risk for adverse pregnancy outcomes [28]. Pearlstone and Baxi reviewed 14 studies from English literature for determining SCH incidence during pregnancy; this incidence varied greatly ranging from 4% to 48%. Small SCHs tend to be more common in the first trimester and appear to pose no added risk to the ongoing pregnancy. Conversely, SCHs in the second trimester often are larger and may be associated with increased risk of preterm delivery [29]. In our study, the SCH incidence was 1.7%, which was lower than that reported in the literature. This may be because some obstetric patients, especially those presenting with vaginal bleeding, are examined in the emergency clinic. Özkaya et al. found subchorionic haemorrhage to be significantly associated with increased risk of miscarriage and intrauterine growth restriction (IUGR) [30]. Therefore, we consider that diagnosing subchorionic haemorrhage in first trimester ultrasonography is important for pregnancy followup. 

The overall incidence of mullein anomalies in the general population is often quoted to be between 2% and 3% [31]. Congenital uterine anomalies are often incidentally discovered in the workup for common obstetrical complications and gynaecologic complaints. Often, mullein anomaly patients do not present in childhood or adolescence but rather in adulthood, when repeated pregnancy loss, persistent menstrual irregularities, or issues related to fertility lead to an unexpected diagnosis. Uterine anomalies have been associated with an increased incidence of spontaneous abortion, malpresentation, placental abruption, IUGR, prematurity, operative delivery, retained placenta, and foetal mortality [32, 33]. We determined uterine anomalies in 0.3% of our pregnant population. Based on the increased incidence of adverse outcomes, we routinely recommend such patients to continue their antenatal care in our high-risk pregnancy clinic. Early first trimester ultrasonography, that is, before 11 weeks of gestation, can help in the early identification of such patients, enabling better care and followup in order to avoid pregnancy loss associated with congenital and acquired uterine anomalies [34].

From a population-based study, Sheiner et al. suggested that the following conditions were significantly associated with uterine leiomyomas: nulliparity, chronic hypertension, hydramnios, diabetes mellitus, and advanced maternal age [35]. In our study, the incidence of uterine fibroids with pregnancy was 0.3%, which correlated with previous reports [36]. No statistical difference was noted between patients aged of below and above 35 years with respect to the presence of uterine masses. This may have been because of the limited number of pregnancies (*n* = 4) complicated with uterine fibroids. Similar to pregnancies with mullerian anomalies, pregnancies with uterine fibroids are followed up in our high-risk pregnancy clinic. Identifying a pregnancy with uterine fibroids in the first trimester can avoid complications such as first trimester bleeding, anaemia during pregnancy, labour dystocia, retained placenta, and the need for neonatal paediatric intensive care [36]. 

Ovarian cysts were noted in 0.7% of our patients (*n* = 9). A retrospective study in Turkey identified pregnancies with adnexal masses requiring surgery over a 6-year period at the Selcuk University Hospital, a tertiary referral centre, between June 2000 and June 2006 and detected 36 such pregnancies from _36_ patients surveyed with a mean age of 26.6 years (range, 18–42 years) [37]. In postoperative histopathology, functional ovarian cysts (41.1%, *n* = 14) were the leading pathologic diagnosis [37]. This does not indicate that all adnexal masses during pregnancy require surgical treatment; however, this study identified the most common pathologic diagnosis of adnexal masses in the Turkish pregnant population. Surgical treatment of persistent adnexal masses in pregnancy, particularly those with a sonographic appearance of a complex tumour (such as tumour >5 cm or characteristic sonographic appearance), is justified because of the high risk of torsion, rupture, and malignancy [38]. Ribic-Pucelj et al. indicated that laparoscopic surgery and surgery in the first trimester do not impair pregnancy outcomes [38]. With these data, it is obvious that first trimester ultrasonographic diagnosis of an ovarian mass and its management can affect the pregnancy outcome.

In our study, 14.7% of our patients had an abnormal pregnancy, 2.9% had a complicated pregnancy, and 0.9% had multiple pregnancy that required careful antenatal care. A total of 185 patients underwent antenal ultrasonography before 11 weeks of gestation. This diagnosis will impact their future antenatal followup.

## 5. Conclusion

The use of ultrasonography in very early stages of pregnancy enables to confirm the status of intrauterine living embryo or diagnose extrauterine pregnancy for which medical treatment with low morbidity is feasible with early detection [39]. First trimester ultrasonography before 11 weeks of gestation is a valuable tool for predicting pregnancy outcomes, particularly with respect to detecting complicated or unviable pregnancies with the exception of aneuploidy. Asymptomatic women with a history of recurrent miscarriage or other risk factors for early pregnancy loss can benefit from an early ultrasonography to assess the viability of the current gestation. Moreover, women who clinically present with threatened abortion but lack appropriate ultrasonography landmarks at a given gestational age may raise suspicion for an ectopic pregnancy; clinical management of such patients may therefore be altered [40]. In our centre, we recommend all pregnant women to undergo first trimester ultrasonography before 11 weeks of gestation; the abovementioned reasons clearly demonstrate the value of this examination. In our study, approximately every 1 of 5 patients benefitted from first antenatal ultrasonography before 11 weeks of gestation. Detecting abnormal and complicated pregnancies as early as possible can prevent a delay in diagnosis and treatment, thus enhancing both maternal and foetal health [39].

## Figures and Tables

**Table 1:** The ultrasonographic diagnosis and their percentage of the patients and their distribution according to age (>35 and ≤35 years).

Ultrasonography (USG)			
	Age (years)		
	≤35	>35	Total
Normal pregnancy	813 (67.1%)	62 (73.8%)	875 (67.5%)
Second USG control patients	213 (17.6%)	13 (15.5%)	226 (17.5%)
Normal pregnancy after USG control	171 (14.1%)	9 (10.7%)	180 (13.8%)
Anembryonic pregnancy after USG control	42 (3.5%)	4 (4.8%)	46 (3.5%)
Anembryonic pregnancy	44 (3.6%)	1 (1.2%)	45 (3.5%)
Intrauterine ex-fetus	50 (4.1%)	4 (4.8%)	54 (4.2%)
Subchorionic hematoma	21 (1.7%)	—	21 (1.6%)
Impaired gestational sac	36 (3.0%)	1 (1.2%)	37 (2.9%)
Uterine anomaly	3 (0.2%)	1 (1.2%)	4 (0.3%)
Ectopic pregnancy	8 (0.7%)	—	8 (0.6%)
Multiple pregnancy	12 (1.0%)	—	12 (0.9%)
Fibroid	3 (0.2%)	1 (1.2%)	4 (0.3%)
Ovarian cysts	8 (0.7%)	1 (1.2%)	9 (0.7%)

Total	1211 (100%)	84 (100%)	1295 (100%)

**Table 2:** The median ages and the median gestational week (days) at the time of diagnosis for each group.

	Age	Gestational period (days)
Normal pregnancy	24.00	56.00
Second USG control patients		
Normal pregnancy after USG control	25.00	42.00
Anembryonic pregnancy after USG control	23.00	45.50
Anembryonic pregnancy	24.00	49.00
Intrauterine ex fetus	23.00	64.00
Subchorionic hematoma	24.00	54.50
Impaired gestational sac	23.00	43.00
Uterine anomaly	28.00	44.00
Ectopic pregnancy	26.00	44.00
Multiple pregnancy	23.00	51.50
Fibroid	29.00	46.00
Ovarian cysts	26.00	46.00

Total	24.00	45.67

*P* value	0.651	

**Table 3:** The statistical difference of the median gestational weeks (days) of ultrasonographic diagnosis.

	Gestational period (days)	*P* value
Normal pregnancy	56.00	
Second USG control patients		0.0001
Normal pregnancy after USG control	42.00	
Anembryonic pregnancy after USG control	45.50	0.0001
Anembryonic pregnancy	49.00	
Intrauterine ex fetus	64.00	
Subchorionic hematoma	54.50	<0.05
Impaired gestational sac	43.00	
Uterine anomaly	44.00	
Ectopic pregnancy	44.00	
Multiple pregnancy	51.50	>0.05
Fibroid	46.00	
Ovarian cysts	46.00	

Total	45.67	

## References

[1] Sohaey R, Woodward P, Zwiebel WJ (1996). First-trimester ultrasound: the essentials. *Seminars in Ultrasound CT and MRI*.

[2] Morin L, Van den Hof MC Ultrasound evaluation of first trimester pregnancy complications.

[3] Nyberg DA, Hill LM, Patterson AS (1992). Normal early intrauterine pregnancy: sonographic development and HCG correlation. *Transvaginal Ultrasound*.

[4] Fossum GT, Davajan V, Kletzky OA (1988). Early detection of pregnancy with transvaginal ultrasound. *Fertility and Sterility*.

[5] Cacciatore B, Tiitinen A, Stenman UH, Ylostalo P (1990). Normal early pregnancy: serum hCG levels and vaginal ultrasonography findings. *British Journal of Obstetrics and Gynaecology*.

[6] Bernard KG, Cooperberg PL (1985). Sonographic differentiation between blighted ovum and early viable pregnancy. *American Journal of Roentgenology*.

[7] Jauniaux E, Johns J, Burton GJ (2005). The role of ultrasound imaging in diagnosing and investigating early pregnancy failure. *Ultrasound in Obstetrics and Gynecology*.

[8] Demianczuk NN, van den Hof MC The use of first trimester ultrasound.

[9] Dighe M, Cuevas C, Moshiri M, Dubinsky T, Dogra VS (2008). Sonography in first trimester bleeding. *Journal of Clinical Ultrasound*.

[10] Bennett GL, Bromley B, Lieberman E, Benacerraf BR (1996). Subchorionic hemorrhage in first-trimester pregnancies: prediction of pregnancy outcome with sonography. *Radiology*.

[11] Nagy S, Bush M, Stone J, Lapinski RH, Gardó S (2003). Clinical significance of subchorionic and retroplacental hematomas detected in the first trimester of pregnancy. *Obstetrics and Gynecology*.

[12] Pedersen JF, Mantoni M (1990). Prevalence and significance of subchorionic hemorrhage in threatened abortion: a sonographic study. *American Journal of Roentgenology*.

[13] Lev-Toaff AS, Coleman BG, Arger PH (1987). Leiomyomas in pregnancy: sonographic study. *Radiology*.

[14] Fedele L, Dorta M, Brioschi D, Giudici MN, Candiani GB (1989). Pregnancies in septate uteri: outcome in relation to site of uterine implantation as determined by sonography. *American Journal of Roentgenology*.

[15] Barber HR (1981). Fetal and neonatal effects of cytotoxic agents. *Obstetrics & Gynecology*.

[16] Nicolaides KH, Heath V, Liao AW (2000). The 11–14 week scan. *Bailliere’s Best Practice and Research in Clinical Obstetrics and Gynaecology*.

[17] Cicero S, Longo D, Rembouskos G, Sacchini C, Nicolaides KH (2003). Absent nasal bone at 11–14 weeks of gestation and chromosomal defects. *Ultrasound in Obstetrics and Gynecology*.

[18] Wisser J, Dirschedl P, Krone S (1994). Estimation of gestational age by transvaginal sonographic measurements of greatest embryonic length in dated human embryos. *Ultrasound in Obstetrics & Gynecology*.

[19] Sepulveda W, Sebire NJ, Hughes K, Kalogeropoulos A, Nicolaides KH (1997). Evolution of the lambda or twin-chorionic peak sign in dichorionic twin pregnancies. *Obstetrics and Gynecology*.

[20] Noble PL, Snijders RJM, Abraha HD, Sherwood RA, Nicolaides KH (1997). Maternal serum free *β*-hCG at 10 to 14 weeks of gestation in trisomic twin pregnancies. *British Journal of Obstetrics and Gynaecology*.

[21] Sezer SD, Küçük M, Yüksel H (2011). Perinatal and neonatal outcomes of twin pregnancies in Turkey. *Twin Research and Human Genetics*.

[22] Fleischer AC, Pennell RG, McKee MS (1990). Ectopic pregnancy: features at transvaginal sonography. *Radiology*.

[23] Dashefsky SM, Lyons EA, Levi CS, Lindsay DJ (1988). Suspected ectopic pregnancy: endovaginal and transvesical US. *Radiology*.

[24] Turner LA, Cyr M, Kinch RAH (2002). Under-reporting of maternal mortality in Canada: a question of definition. *Chronic Diseases in Canada*.

[25] Soussis I, Dimitry ES, Oskarsson T, Margara R, Winston R (1991). Diagnosis of ectopic pregnancy by vaginal ultrasonography in combination with a discriminatory serum hCG level of 1000 IU/l (IRP) [comment on British Journal of Obstetrics and Gynaecology, vol. 97, pp. 904–908, 1990]. *British Journal of Obstetrics and Gynaecology*.

[26] Fleischer AC, Pennell RG, McKee MS (1990). Ectopic pregnancy: features at transvaginal sonography. *Radiology*.

[27] Bakken IJ, Skjeldestad FE (2006). Time trends in ectopic pregnancies in a Norwegian county 1970–2004—a population-based study. *Human Reproduction*.

[28] Nagy S, Bush M, Stone J, Lapinski R, Gardó S (2005). Clinical significance of subchorionic and retroplacental hematomas detected in the first trimester of pregnancy. *Orvosi Hetilap*.

[29] Pearlstone M, Baxi L (1993). Subchorionic hematoma: a review. *Obstetrical and Gynecological Survey*.

[30] Özkaya E, Altay M, Gelişen O (2011). Significance of subchorionic haemorrhage and pregnancy outcome in threatened miscarriage to predict miscarriage, pre-term labour and intrauterine growth restriction. *Journal of Obstetrics and Gynaecology*.

[31] Sanfilippo JS, Wakim NG, Schikler KN, Yussman MA (1986). Endometriosis in association with uterine anomaly. *American Journal of Obstetrics and Gynecology*.

[32] Cohen AW, Chhibber G (1986). Obstetric complications of congenital anomalies of the paramesonephric ducts. *Seminars in Reproductive Endocrinology*.

[33] Green LK, Harris RE (1976). Uterine anomalies. Frequency of diagnosis and associated obstetric complications. *Obstetrics and Gynecology*.

[34] Devi Wold AS, Pham N, Arici A (2006). Anatomic factors in recurrent pregnancy loss. *Seminars in Reproductive Medicine*.

[35] Sheiner E, Bashiri A, Levy A, Hershkovitz R, Katz M, Mazor M (2004). Obstetric characteristics and perinatal outcome of pregnancies with uterine leiomyomas. *Journal of Reproductive Medicine for the Obstetrician and Gynecologist*.

[36] Kellal I, Haddouchi NE, Lecuyer AI, Body G, Perrotin F, Marret H Leiomyoma during pregnancy: which complications?. *Obstetrics, Gynecology & Infertility*.

[37] Balci O, Gezginc K, Karatayli R, Acar A, Celik C, Colakoglu MC (2008). Management and outcomes of adnexal masses during pregnancy: a 6-year experience. *Journal of Obstetrics and Gynaecology Research*.

[38] Ribic-Pucelj M, Kobal B, Peternelj-Marinsek S (2007). Surgical treatment of adnexal masses in pregnancy: indications, surgical approach and pregnancy outcome. *Journal of Reproductive Medicine*.

[39] Sen C (2001). The use of first trimester ultrasound in routine practice. *Journal of Perinatal Medicine*.

[40] Dulay AT, Copel JA (2008). First-trimester ultrasound: current uses and applications. *Seminars in Ultrasound, CT and MRI*.

